# A closer look reveals hidden diversity in the intertidal Caribbean Fortuyniidae (Acari, Oribatida)

**DOI:** 10.1371/journal.pone.0268964

**Published:** 2022-06-15

**Authors:** Tobias Pfingstl, Sylvia Schäffer, Iris Bardel-Kahr, Julia Baumann

**Affiliations:** Institute of Biology, Karl-Franzens University of Graz, Graz, Austria; Nanjing Agricultural University, CHINA

## Abstract

A molecular genetic and morphometric investigation revealed the supposedly widespread Caribbean and Western Atlantic intertidal oribatid mite species *Fortuynia atlantica* to comprise at least two different species. Although there are no distinct morphological differences separating these taxa, *COI* and *18S* sequence divergence data, as well as different species delimitation analyses, clearly identify the two species. *Fortuynia atlantica* is distributed in the northern Caribbean and the Western Atlantic and the new *Fortuynia antillea* sp. nov. is presently endemic to Barbados. Vicariance is supposed to be responsible for their genetic diversification and stabilizing selection caused by the extreme intertidal environment is suggested to be the reason for the found morphological stasis. The genetic structure of *Fortuynia atlantica* indicates that Bermudian populations are derived from the northern Caribbean and thus support the theory of dispersal by drifting on the Gulf Stream. Haplotype network data suggest that Bermudian and Bahamian populations were largely shaped by colonization, expansion and extinction events caused by dramatic sea level changes during the Pleistocene. A preliminary phylogenetic analysis based on *18S* gene sequences indicates that the globally distributed genus *Fortuynia* may be a monophyletic group, whereas Caribbean and Western Atlantic members are distinctly separated from the Indo-Pacific and Western Pacific species.

## Introduction

Oribatid mites are small arachnid organisms that have occupied nearly every ecological niche in terrestrial environments from soil, litter, lichen etc. to the canopy of trees. Intertidal oribatid mites are exceptional among this diverse group because they only occur in the marine littoral area and have adapted to daily tidal flooding. Although their morphology is basically conforming to that of typical terrestrial oribatid mites, they show significantly longer claws to cope with tidal wave action [1] and they exhibit elaborate plastron respiration systems to breathe underwater [2, 3]. In the littoral environment, they dwell in a wide range of habitats, as for example rocky cliffs, boulder beaches, muddy sand, mangrove forests or manmade concrete structures, where they feed on diverse intertidal algae [4]. On subtropical and tropical coasts members of only two intertidal families can be found, namely the Fortuyniidae and Selenoribatidae.

The Fortuyniidae comprise four genera, *Alismobates*, *Circellobates*, *Fortuynia* and *Litoribates*, whereas *Fortuynia* with presently 17 known species is the most diverse taxon of this family. Members of this genus are distributed worldwide, but most species and findings were reported from the Indo-Pacific area [5]. Reports from the Caribbean, on the other hand, were initially scarce and provided only vague information about the occurrence of undetermined *Fortuynia* species from Barbados and the Dominican Republic [5]. A recent paper [6] provided a more comprehensive insight and reported occurrences of a specific *Fortuynia* species, namely *Fortuynia atlantica*, from Grenada, Guadeloupe, Florida and the Bahamas. This species was originally discovered on the small and remote archipelago of Bermuda in the western Atlantic and it was the first species of this genus and family showing an obvious sexual dimorphism [7]. Males possess several leaf-like modified notogastral setae and a pair of remarkable lateral protuberances on their posterior gastronotic region [7]. Though it was suggested that this dimorphism may be related to some kind of mating behaviour, observations or any other evidence are lacking so far [8]. Apart from this unusual morphology, the geographic origin of this species was also a matter of conjecture. Due to the young geological age of Bermuda, it was assumed that *F*. *atlantica* is derived from populations somewhere in the Caribbean and experiments demonstrated that these mites would theoretically be able to survive transport on the sea surface from these allegedly areas of origin to Bermuda via the Gulf Stream [9]. The recent reports of *F*. *atlantica* on the Bahamas and Florida support this theory [6] and point to a wider Caribbean distribution of this species but molecular genetic data linking all these populations is yet missing.

However, based on its complex geological history, the Caribbean is known to show high levels of endemism and only a minority is represented by widespread species, presumably taxa with good dispersal abilities [10]. Consequently, allegedly widespread Caribbean taxa with poor dispersal abilities are likely to contain hidden species complexes. In fact, there are many cases known from the Caribbean supporting this statement. For example, the orb-weaver spider *Micrathena* with six nominal species consists in fact of eight divergent genetic lineages probably representing all single island endemics [11] or the skipper butterfly *Astraptes fulgerator*, which has long been regarded as a single species, turned out to contain at least three different species [12]. Moreover, a recent study [13] demonstrated a similar case of cryptic diversity in two Caribbean intertidal oribatid mites. *Carinozetes bermudensis* and *Carinozetes mangrovi*, both species originally described from Bermuda, just like *F*. *atlantica*, were found to be widespread on Caribbean coasts whereas a molecular genetic investigation revealed them to consist of five distinct genetic lineages, probably representing different species. Furthermore, the allegedly widespread *Thalassozetes barbara* [6], another intertidal mite species, was shown to consist of at least seven different species with nearly each representing a Caribbean island endemic [14].

In view of this, the trans-Caribbean occurrence of a single tiny wingless *Fortuynia atlantica* species is questionable and should be further evaluated. For this purpose, we investigated several populations from different Caribbean and Bermudian islands or landmasses by means of morphometric and molecular genetic analyses to clarify whether they represent a single widespread species with unexpected dispersal abilities or various species, lineages respectively, showing strikingly similar morphologies. Additionally, we intended to (i) update biogeographic knowledge about Caribbean intertidal mites and knowledge about their dispersal abilities and to (ii) provide first phylogenetic data of available members of the genus *Fortuynia*.

## Materials and methods

### Sample collection

In the years 2011 and 2016–2018, coastal mites were collected during three fieldtrips to Bermuda and several regions in the Caribbean. Samples of intertidal algae were scraped off rocks with a knife during low tide. Algae were then put in Berlese-Tullgren funnels for about 24 hours to extract mites. Collected specimens were stored in ethanol (100%) for morphological and molecular genetic investigation. Samples from Grenada and Guadeloupe contained each only a single specimen and these were used to make microscopic preparations and thus are not included in morphometric and molecular genetic analyses. Samples from 2011 were all collected by T. Pfingstl and samples from 2016–2018 by T. Pfingstl and A. Lienhard.

### Sample locations

1. Bermuda: Gunner Bay (BE_30; coordinates: 32.372157–64.653853) brown alga from rocks, median eulittoral, 11 Sep. 2011. Whalebone Bay (BD_06; coordinates: 32.365384–64.713798) *Gardnerula* (intertidal alga) on rocks, upper eulittoral; (BD_08; coordinates: 32.365184, -64.713117) diverse algae on rocks, 23 May 2018.
2. U.S.A.: Florida, Florida Keys, Islamorada (FL_18; coordinates: 24.9378169–80.612182) *Bostrychia* (intertidal alga) on rocks, median eulittoral, 13 Feb. 2017.
3. Bahamas, New Providence Island: Goodman Bay (BH_12; coordinates: 25.071769–77.384046) *Bostrychia* on rocks, median eulittoral, 19 Feb. 2017. South Beach (BH_13; coordinates: 25.007377–77.339388) *Bostrychia* on rocks next to mangroves, lower to median eulittoral; (BH_14; coordinates: 25.001339–77.350229) diverse algae from mangrove roots, lower eulittoral, 20 Feb. 2017. Jaws Beach (BH_19; coordinates: 25.018155–77.546636) *Bostrychia* from flat rocks, lower eulittoral; (BH_20; coordinates: 25.018154–77.546637) *Gardnerula* on rocks, lower eulittoral; 20 Feb. 2017.
4. Barbados: Bridgetown, Carlisle Bay (BA_17; coordinates: 13.079329–59.61255) brown short tomentose alga from quay wall, median eulittoral, 26 Feb. 2017. Bathsheba (BA_28; coordinates: 13.062537–59.541903) *Bostrychia* from rocks, upper eulittoral; 28 Feb. 2017.
5. Guadeloupe, Basse-Terre: Bois Jolan (GU_13; coordinates: 16.237130–61.346978) *Bostrychia* on rocky shore, upper eulittoral, 20 Feb. 2016.
6. Grenada: La Sagesse (GR_10; coordinates: 12.023974–61.671661) *Bostrychia* on rocks, median eulittoral, 27 Feb. 2016.

### Morphometric analyses

For morphometric studies, specimens were placed in lactic acid (temporary slides) and measurements were performed using a compound light microscope (Olympus BH-2) and ocular micrometer. Individuals used for morphometric studies were not the same as used for molecular genetic studies but belonged to the exact same populations (patch of algae ca. 10 cm^2^). The specimens used for molecular genetic analyses were identified belonging to the exact same morphospecies (with the help of a stereomicroscope) prior to their destruction for the extraction of DNA.

**Fig 1:**
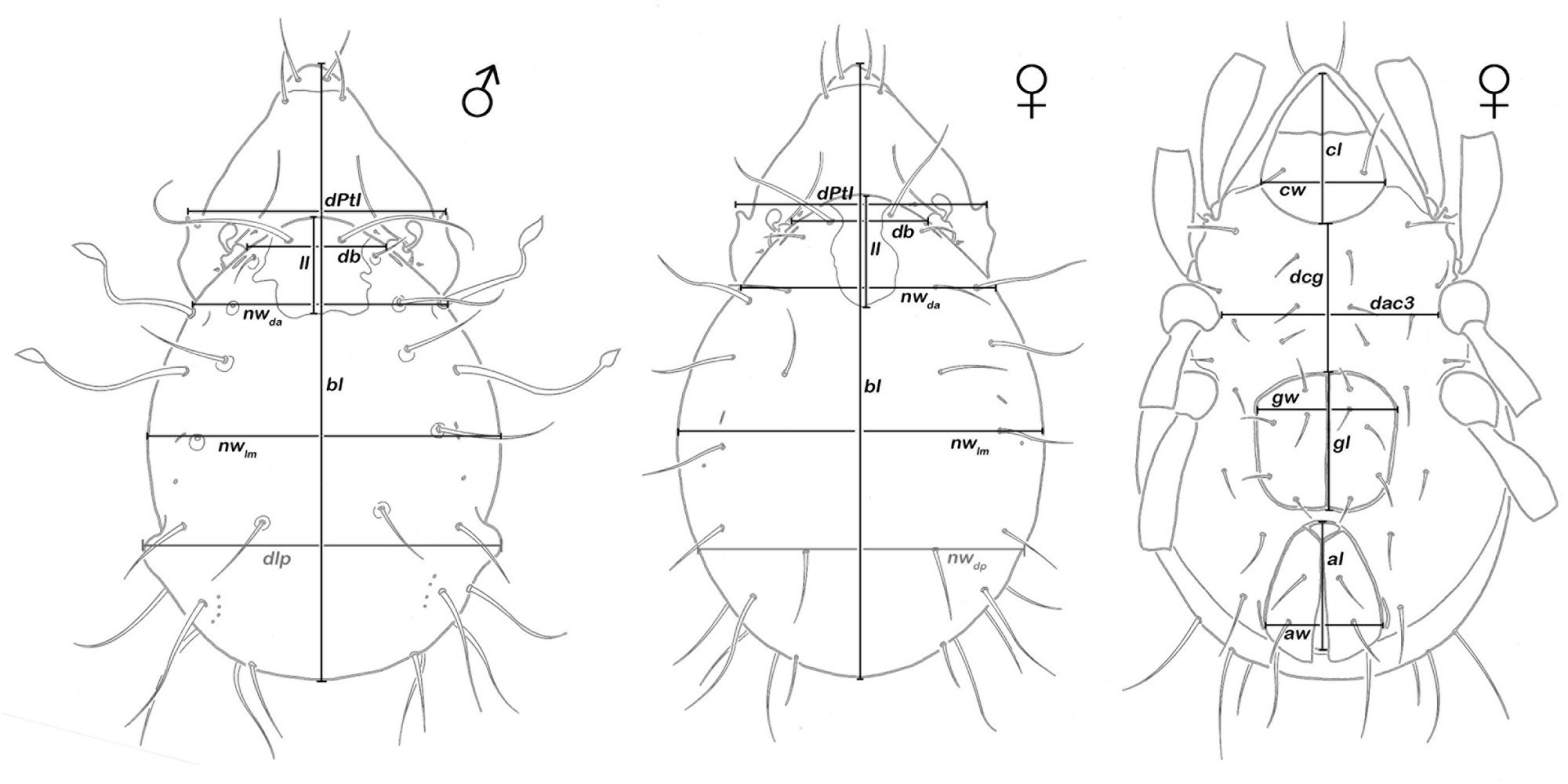
Graphic illustration of a male and a female *Fortuynia atlantica* showing the remarkable sexual dimorphism and highlighting measured continuous dorsal and ventral variables. Dorsal aspect (left and center): ***bl***–body length, ***dPtI***–distance between pedotecta I, ***db***–distance between bothridia, ***ll***–lenticulus length, ***nw***_***da***_−notogastral width on level of seta *da*, ***nw***_***lm***_−notogastral width on level of seta *lm*, ***dlp***–distance between lateral protuberances (only males), ***nw***_***dp***_−notogastral width on level of seta *dp* (only in females). Ventral aspect: ***cl***–camerostome length, ***cw***–camerostome width, ***dcg***–distance between camerostome and genital orifice, ***dac3*** –distance between acetabula 3, ***gl***–genital orifice length, ***gw***–genital orifice width, ***al***–anal opening length, ***aw***–anal opening width. (Sexually dimorphic variables shown in weaker color).

A total of 16 continuous variables (Fig 1) were measured in 90 specimens of *Fortuynia* from Bahamas, Barbados and Bermuda (Bahamas n = 28 / BH_12, BH_13, BH_19, BH_20; Barbados n = 24 / BA_17, BA_28; Bermuda n = 38 / BD_06, BE_30). Populations from a single island were pooled for all morphometric analyses. As the genus displays a distinct sexual dimorphism, affected variables were only measured in the respective gender (i.e. *nwdp* was measured only in females and *dlp* only in males) and only used for comparison between specimens of the same sex. Mean, standard deviation, coefficient of variation (cv), minimum and maximum of each variable were calculated for the three locations. Because the sexual dimorphism also affects the length and width of the genital opening (S3 Fig), the respective variables *gl* and *gw* were treated separately for females and males. Kruskal-Wallis- and Mann-Whitney-U test were performed to compare the means of the variables within all three populations and in pairwise comparisons, respectively.

Sexually dimorphic variables *nwdp*, *dlp*, *gl* and *gw* were excluded from multivariate analyses investigating differences between the three locations. Data was size-corrected as described in [15], and multivariate analyses were performed on ln(x + 1) transformed raw as well as size-corrected data. If not indicated otherwise, analyses were performed with PAST 3.11 [16].

Principal component analysis (PCA) was used to visualize the pattern of variation among the *Fortuynia atlantica* populations. To determine the optimum number of clusters, present in the data, partitioning clustering by k-means was performed with the package *factoextra* [17] implemented in R v. 3.6.2 [18]. The applied gap statistics followed the criterion of [19] with a bootstrap of 500.

As both ordination by PCA and partitioning clustering showed that the sexual dimorphism still had a strong impact on total variation even after removing the abovementioned affected variables, the sexes were subsequently analyzed separately.

Canonical variates analysis (CVA) on groups coded according to sex and populations (thus, the groups were: Bahamas males, Bahamas females, Barbados males, Barbados females, Bermuda males, and Bermuda females) was performed to calculate squared Mahalanobis distances (D^2^), which were used to evaluate whether the differences between the three populations were larger in females or males.

CVA was also conducted on both sexes separately in order to evaluate which variables were contributing most to the differences between the populations and whether the same variables were important in both sexes. The performance of classification by CVA was tested by calculating the number of specimens correctly classified by all-samples CVA and leave-one-out cross-validation (LOO-CV) CVA, and the equality of means of the populations was tested by permutational multivariate analysis of variance (PERMANOVA). The differences in dispersion of each population were evaluated using the functions betadisper and permutest in the R package *vegan* [20].

### Molecular genetic analyses

As specimens used for morphometry and molecular genetic analyses were not the exact same individuals, specimens were checked, before DNA extraction, under a stereomicroscope to confirm that they belong to the exact same morphospecies found at the respective location. Whole genomic DNA was extracted from 71 ethanol-fixed specimens using Chelex resin as described in [21], with minor adjustments: Whole specimen were crushed in microcentrifuge tubes which contained 50 μL Chelex solution and 1.7 μL Proteinase K. For this study two gene fragments were sequenced: the mitochondrial cytochrome c oxidase subunit 1 gene (*COI*) and the nuclear *18S* rRNA gene (*18S*). Region 2 of *COI* (around 590 bp) was amplified using the primers Mite *COI*‐2F and Mite *COI*‐2R [22] with a 2-step PCR. Reaction volume was 10.5 μL, containing 1 μL 10x reaction buffer, 1 μL of each primer (10 mM), 1 μL dNTP mix (10 mM), 0.75 μL MgCl_2_ (25 mM), 0.05 μL of Taq polymerase (5 units μl^−1^, BioThermRed), 3.5 μL deionized water, and 2 μL of DNA template. The program consists of a denaturation step at 94°C for 5 min, followed by 18 cycles of the first step (denaturation for 30 sec at 94°C, annealing for 30 sec at 47°C, extension for 1 min at 72°C), then 22 cycles of the second step (denaturation for 30 sec at 94°C, annealing for 30 sec at 50°C, extension for 1 min at 72°C), ending with a final extension step at 72°C for 7 min. The complete *18S* gene (~ 1.8 kbp) was amplified in overlapping fragments using the primer pairs *18S*fw/rev960 (I) and fw390/*18S*rev (II) [23] in reaction volumes of 10.5 μL (same composition as *COI*-2). PCR conditions were 94°C for 5 min, then 45 cycles of denaturation (94°C, 30 sec), annealing (46.5°C, 30 sec), extension (72°C, 1 min), followed by a final extension step at 72°C for 7 min. Subsequent DNA purification steps included enzymatic ExoSAPIT (Affymetrix) and Sephadex G-50 resin (GE Healthcare). Cycle sequencing, using BigDye Sequence Terminator v3.1 kit (Applied Biosystems), was conducted in 10 μL reaction volume according to the protocol by ThermoFisher. The conditions in the thermocycler were 3 min at 94°C, followed by 35 cycles of denaturation (94°C, 30 sec), annealing (50°C, 30 sec), extension (60°C, 4 min), with a final extension step of 60°C for 7 min. For *COI*-2 the aforementioned primers were used. For the *18S* gene the primers rev480, fw390 (I), fw770 and fw1230 (II) ([23] were used. Automatic capillary sequencing and sequence visualization was operated on an ABI3500XL (Applied Biosystems) device.

### Phylogenetic analyses

The standard *COI* (s*COI*-2) and *18S* (s*18S*) datasets comprised 74 fortunyiid sequences (71 ingroup/*Fortuynia* and 3 outgroup taxa/*Alismobates*) all generated in the present study, except for 3 *Fortuynia COI*-2 sequences taken from GenBank. To study the phylogenetic placement of the herein studied species, we further combined 22 *18S* sequences of Ameronothroidea from GenBank with nine sequences from this study (following the results of the *COI*-2 analyses, two of each mt lineage and the three outgroup *Alismobates*) in one alignment (p*18S*; for details see S1 Table). *18S* sequences were aligned by MAFFT [24] using -auto strategy and normal alignment mode. Unambiguous sites were trimmed in trimAl [25] using automated1 command.

All phylogenetic analyses were performed on the platform PhyloSuite v.1.2.2 [26]. ModelFinder [27] was used to select the best substitution model, which was according to Bayesian information criterion HKY+F+I for both s*COI* and s*18S*, and K2P+I+G4 for p*18S*. Maximum-likelihood (ML) trees were constructed using IQ-tree [28] under the best substitution model for 5000 ultrafast [29] bootstraps, as well as the Shimodaira–Hasegawa–like approximate likelihood-ratio test (SH-aLRT; [30]). The Bayesian inference (BI) trees were inferred using MrBayes 3.2.6 [31] under the same models as stated above. We performed two parallel runs with four chains, each for 10 million generations sampled every 1,500th generation. The initial 25% of sampled data were discarded as burn-in.

TCS network [32] in the software PopArt [33] was used to construct haplotype networks of all 68 fortuyniid specimens (excluding outgroup taxa/3 *Alismobates*).

For species delimitation analyses (SDA) based on *COI*-2 data, we employed several methods: the “Assemble Species by Automatic Partitioning” (ASAP) method [34] and the two tree-based approaches “Generalized Mixed Yule Coalescent” (GMYC [35], and the ML partition “Poison Tree Process”(PTP-ML) model. The applied settings and programs/packages for the SDA analyses followed [36], except for ASAP applying default parameters and uncorrected p-distances. BEAST2 [37] was used to obtain ultrametric tree(s) which is/are the input needed for GMYC and the Bayesian version of GMYC (bGMY [38]), analyses. Settings therefore were as follows: relaxed log normal, birth death model, chain length 400 million generations, sampling every 2,500th generation. Resulting log-files were evaluated in Tracer v.1.7 [39] and after discarding the initial 50% of trees as burn-in, remaining 80,001 treefiles were used for a maximum clade credibility (MCC) tree. For bGMYC analysis, trees from the Beast runs were resampled at lower frequency, obtaining a final dataset with 400 trees.

All generated sequences were deposited at GenBank and are accessible at following numbers, ON239327–ON239397 (*COI*) and ON243678–ON243686 (*18S*).

### Microscopic investigations

Specimens were embedded in Berlese mountant for microscopic investigation in transmitted light and these preparations were studied using an Olympus BH-2 Microscope.

For photographic documentation, specimens were air-dried and photographed using a Keyence VHX-5000 digital microscope with automated image stacking.

### Nomenclatural acts

The electronic edition of this article conforms to the requirements of the amended International Code of Zoological Nomenclature, and hence the new names contained herein are available under that Code from the electronic edition of this article. This published work and the nomenclatural acts it contains have been registered in ZooBank, the online registration system for the ICZN. The ZooBank LSIDs (Life Science Identifiers) can be resolved and the associated information viewed through any standard web browser by appending the LSID to the prefix "http://zoobank.org/". The LSID for this publication is: urn:lsid:zoobank.org:pub:68576E45-5195-4FF9-9B85-6F0E6A32B0F2. The electronic edition of this work was published in a journal with an ISSN, and has been archived and is available from the following digital repositories: PubMed Central, LOCKSS

## Results

### Morphometric data

Univariate Statistics–The variability of all characters as indicated by its cv was moderate (< 0.10) in all three populations (Table 1). In 72.2% of the variables, at least one of the three populations differed significantly from the others. Pairwise comparisons showed that in most of these variables a significant difference between the specimens from Barbados and at least one of the other two populations (Bahamas, Bermuda) was present. An exception was found in the length of the genital plate *gl* for females, which differed significantly only between Bahamas and Bermuda.

**Table 1:** Mean (x), standard deviation (sd), coefficient of variation (cv), minimum (min) and maximum (max) of three populations of *Fortuynia atlantica*. Results of Kruskal-Wallis and Mann–Whitney-U test are given; *p < 0.05, **p < 0.01, ***p < 0.001. a Bermuda vs Bahamas, b Bermuda vs Barbados, c Bahamas vs Barbados, d all possible combinations.

	Bahamas (n = 28; 14m/14f)				Barbados (n = 24; 12m/12f)				Bermuda (n = 38; 11m/27f)				Kruskal-Wallis	Mann-Whitney-U
	x	cv	min	max	x	cv	min	max	x	cv	min	max		
*bl*	421	0.03	394	444	421	0.02	406	439	438	0.02	419	456	***	a, b ***
*dPtI*	172	0.02	166	179	177	0.03	160	185	176	0.02	166	183	***	a, b ***
*db*	92	0.03	86	95	97	0.05	80	105	92	0.04	83	99	***	b, c ***
*ll*	70	0.09	62	80	75	0.07	65	83	76	0.09	62	89	**	b, c **
*nw*_*da*_	180	0.06	160	197	178	0.06	160	194	186	0.07	157	206	*	b *
*nw*_*lm*_	242	0.05	222	271	239	0.03	222	255	248	0.05	222	274	**	b **
*nw*_*dp*_	231	0.04	219	246	223	0.04	197	234	231	0.05	206	252		
*dlp*	239	0.02	231	249	244	0.02	236	249	244	0.03	228	252		
*cl*	122	0.02	117	126	123	0.02	117	129	123	0.03	117	132		
*cw*	90	0.02	86	95	95	0.02	89	99	92	0.02	89	99	***	d ***
*dcg*	93	0.04	86	99	93	0.04	86	102	97	0.04	89	105	***	a, b **
*dac3*	142	0.03	132	151	147	0.03	139	154	147	0.02	139	154	***	a, c ***
*gl-*female	85	0.04	80	89	86	0.03	80	89	88	0.04	80	95	*	a *
*gw-*female	101	0.04	92	108	101	0.02	97	105	103	0.03	95	108	*	b *
*gl-*male	73	0.04	65	77	75	0.05	71	80	77	0.06	71	83		
*gw-*male	87	0.03	83	89	88	0.02	86	90	89	0.03	83	95		
*al*	91	0.05	83	99	91	0.05	77	99	96	0.06	83	105	***	a **, b ***
*aw*	74	0.06	65	83	73	0.05	68	80	78	0.05	71	83	***	a *, b ***

**Fig 2:**
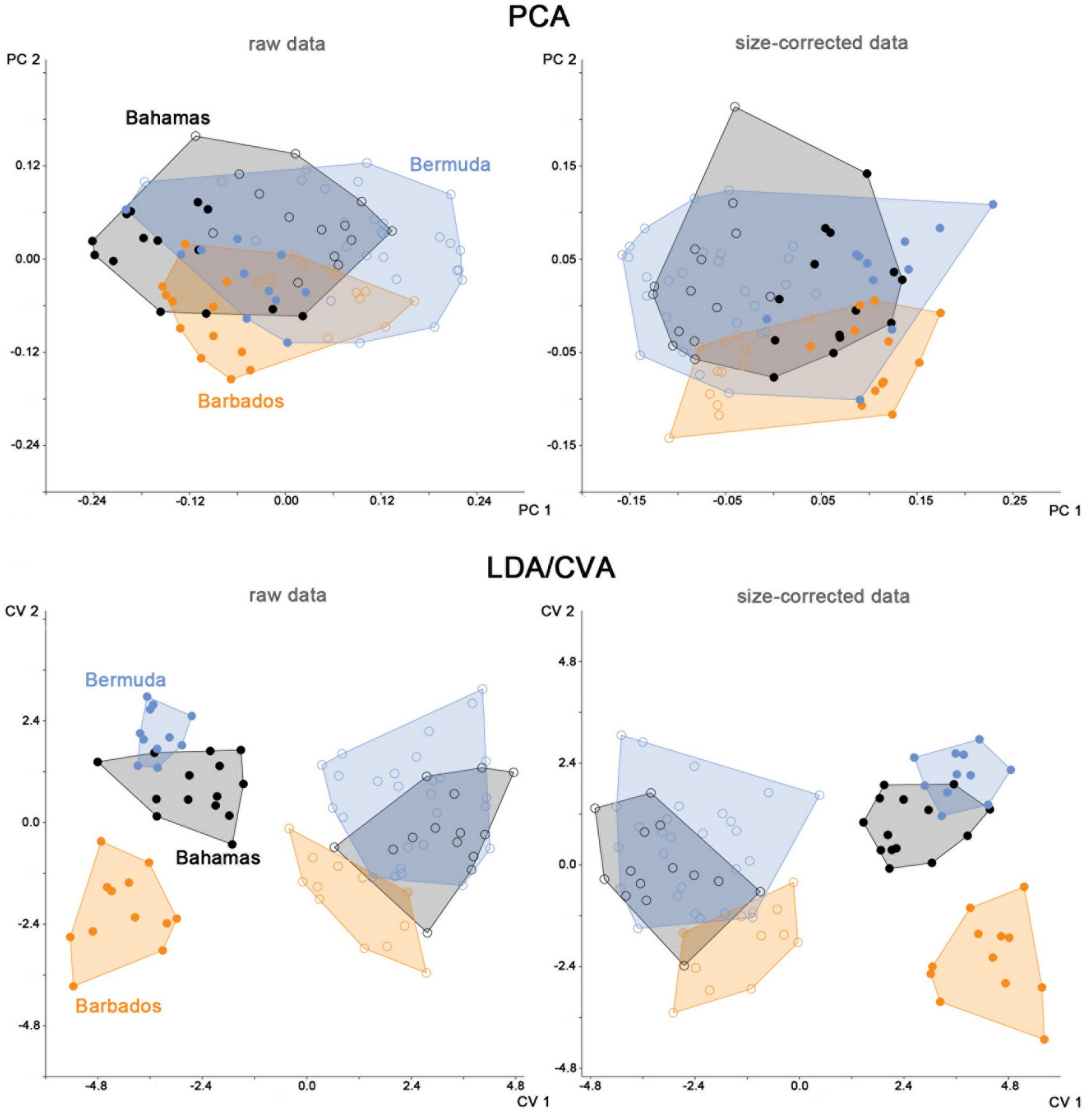
Graph showing results of morphometric analyses of three different populations of *Fortuynia atlantica*. Upper row PCA graphs, lower row LDA/CVA graphs with males and females segregated. Colors refer to geographic locations, full circles represent male and empty circles represent female specimens.

Multivariate Statistics–PCA on both raw and size-corrected data showed that the three populations cluster together. The population from Barbados was slightly separated from the other two populations, but with large overlapping areas (Fig 2), whereas this separation was better recognizable in the size-corrected data. In contrast to the three populations, the two sexes were remarkably separated along PC 1 in both graphs. Partitioning clustering on all specimens supported this observation: gap statistics on k-means clustering revealed that the optimum number of clusters was two in raw as well as in size-corrected data, and the found clusters corresponded to sex. In the raw data, 86% of males and 79% females were correctly assigned to their respective cluster, and in size-corrected data 84% of males and even 92% of females were correctly assigned. The tendency of the data to form clusters still was low in both data sets (Hopkins statistic of 0.61 in both cases). When the two sexes were analyzed separately, no clustering whatsoever could be detected neither in raw nor in size-corrected data (accordingly, Hopkins statistic was in all cases below 0.60, indicating a low clustering tendency).

In the CVA on the three populations coded by sex, the first axis (explaining 72.5% of the total variation in raw and 76.7% in the size corrected data) also separated females and males in raw as well as size corrected data (Fig 2). Visual evaluation of the CVA plot furthermore indicated that the three populations were better separated in males than in females in both data sets, and the calculated values for D^2^ confirm that the distances between the populations are higher in males than in females (Table 2). PERMANOVA showed that every population of females differed significantly (p < 0.01) from every population of males in raw as well as size-corrected data. In the raw data, all differences between populations within each sex also were highly significant, whereas in the size-corrected data no significant differences were present between Bahamas and Bermuda in both sexes (but between all other populations).

**Table 2:** Squared Mahalanobis distances (D^2^) between the populations in raw and size-corrected data of females and males, respectively.

	raw data		size-corrected data	
	female	male	female	male
Bahamas—Barbados	11,1	17,8	11,1	15,2
Bermuda—Barbados	9,4	19,5	8,3	21,0
Bahamas—Bermuda	4,9	11,6	3,3	7,8

The CVAs conducted on females as well as on males separately revealed a separation of the population from Barbados from those from Bahamas and Bermuda on the first canonical axis, which explained most of the total variation in raw as well as size-corrected data (Table 3). In females, the variables contributing most to the variation (defined as the loadings with the first three highest values) along the first axis were *ll*, *nwlm*, *nwdp* and *cw* in raw data and *bl*, *nwlm* and *nwdp* in size corrected data. In males, the respective loadings were *db*, *cw* and *dcg* in raw data and *bl*, *db* and *dcg* in size corrected data.

**Table 3:** Loadings of variables in CVA on raw and size-corrected data of females and males. Loadings with the first three highest values are in bold.

	females				males			
	raw data		size-corrected data		raw data		size-corrected data	
	Axis 1	Axis 2	Axis 1	Axis 2	Axis 1	Axis 2	Axis 1	Axis 2
% of variation explained by axis	58.11	41.89	71.1	28.9	84.48	15.52	86.53	14.47
*bl*	0.007	-0.012	**0.018**	**0.018**	-0.002	**0.013**	**-0.009**	**0.015**
*dPtI*	-0.002	-0.008	-0.004	-0.002	0.003	**0.013**	0.003	0.006
*db*	-0.007	0.005	-0.004	**-0.014**	**0.010**	0.003	**0.007**	-0.004
*ll*	**-0.017**	**-0.020**	-0.011	0.006	0.004	**0.021**	0.002	0.007
*nw*_*da*_	0.007	-0.008	0.009	0.002	-0.001	-0.001	-0.002	**-0.015**
*nw*_*lm*_	**0.010**	-0.006	**0.017**	-0.004	-0.001	0.005	-0.003	**-0.009**
*nw*_*dp*_	**0.011**	-0.002	**0.020**	**-0.010**	0.001	0.007	0.002	-0.005
*cl*	0.003	-0.003	0.003	-0.007	0.002	0.003	0.001	-0.007
*cw*	**-0.010**	-0.006	-0.007	-0.005	0.008	0.009	0.005	0.000
*dcg*	0.001	**-0.018**	-0.001	0.008	**-0.008**	**0.016**	**-0.006**	0.005
*dac3*	-0.007	-0.005	-0.008	-0.008	0.003	0.011	0.003	0.002
*gl*	0.002	-0.013	0.000	0.004	0.000	**0.013**	-0.001	0.003
*gw*	0.004	-0.008	0.002	0.000	0.000	0.006	-0.001	-0.002
*al*	0.006	**-0.018**	0.003	**0.010**	-0.004	0.010	-0.003	0.001
*aw*	0.007	-0.009	0.004	0.001	-0.005	0.010	-0.003	0.000

The percentage of specimens correctly classified by CVA was higher in the raw data compared to size-corrected data in both sexes, and it was always higher in males than in females (Table 4). PERMANOVA performed on females as well as on males showed highly significant (p < 0.001) differences between the means of all three populations in raw data and no significant differences between Bahamas and Bermuda, but between all other populations, in size-corrected data.

**Table 4:** Percentage of specimens correctly classified by all samples CVA and LOO-CV CVA on raw and size-corrected data of females and males, respectively.

	females		males	
	all samples CVA	LOO-CV CVA	all samples CVA	LOO-CV CVA
raw data	98.1%	69.8%	100%	83.8%
size-corrected data	88.7%	60.4%	97.3%	75.6%

In females, the dispersion of the populations from Barbados and Bermuda differed significantly (p < 0.5) in raw data, and in size-corrected data it differed significantly between Barbados and Bermuda and between Barbados and Bahamas. In males, no significant differences in dispersion were found in raw data, but in size-corrected data the dispersion differed significantly between the populations Barbados and Bermuda and between Barbados and Bahamas. In all instances, it was the population from Barbados which showed lower dispersion.

### Molecular genetic data

**Fig 3:**
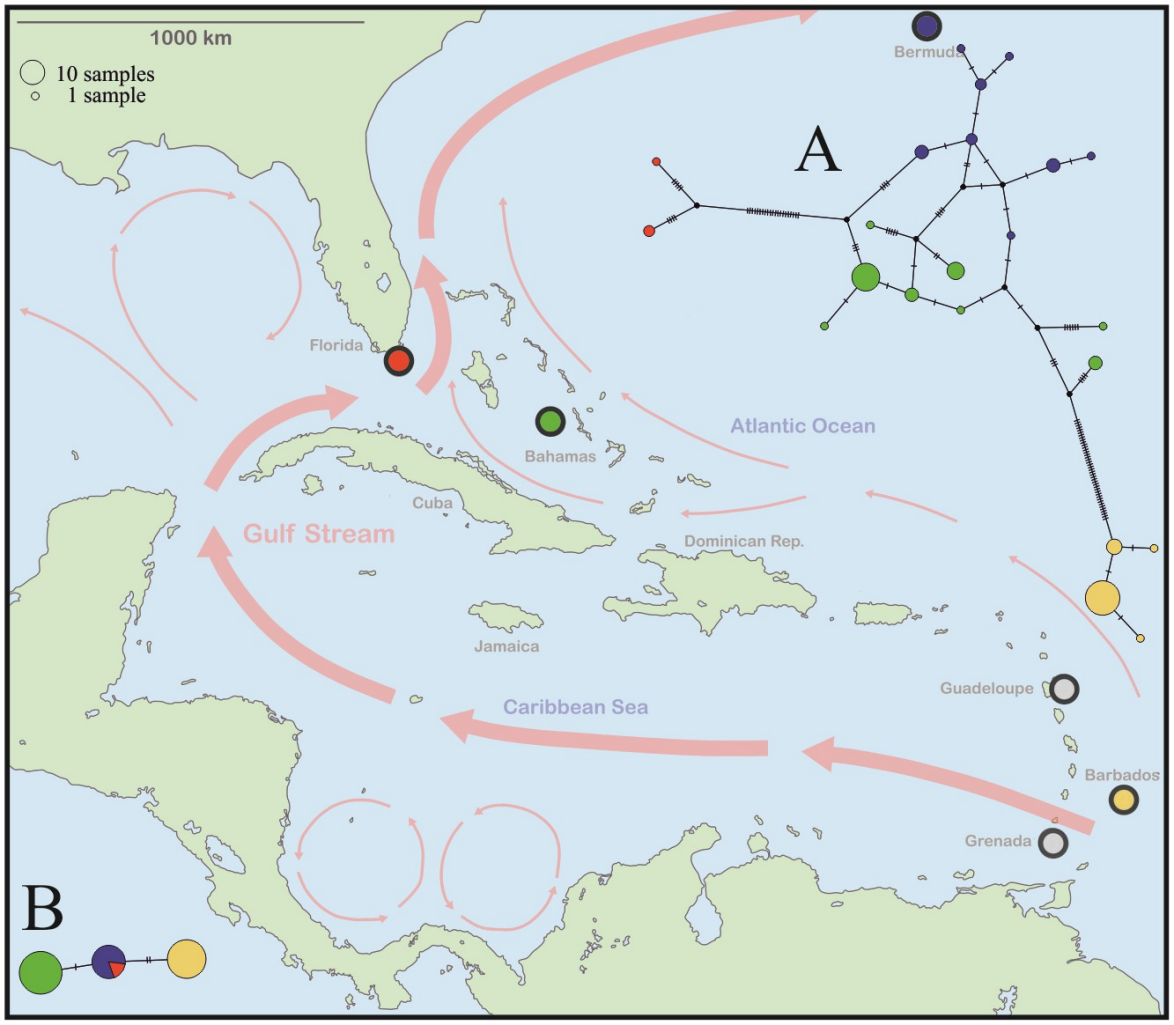
Map showing records of *Fortuynia* populations in the Caribbean and Western Atlantic area. Different colors refer to different locations whereas records given in grey refer to findings of single specimens (not included in morphometric and molecular genetic analyses). Red arrows indicate ocean currents and their directions. Inserts showing haplotype network based on *COI*-2 (A), respectively, *18S* (B) sequences. Colors of haplotypes refer to geographic location.

The *COI*-2 haplotype diversity of herein studied *Fortuynia* populations was relatively high as TCS haplotype analysis identified 22 different haplotypes. These show a clear geographic pattern but with considerable genetic differentiation (Fig 3A). Barbadian specimens show four haplotypes with very few mutations in between, Bermudian and Bahamian populations exhibit eight haplotypes each, with considerable divergence between certain haplotypes of the latter, and the few specimens from Florida show two different haplotypes but these are separated by a conspicuous number of mutations. According to the s*18S* dataset, populations from Barbados and Bahamas show each a single haplotype, while populations of Florida and Bermuda share one same haplotype (Fig 3B).

Phylogenetic reconstructions based on BI and ML revealed highly similar results, therefore, only BI trees are given in S1 and S2 Figs. Both single gene analyses yielded in general poorly resolved phylogenies, congruently supporting only the monophyly of one clade which includes all Barbadian specimens. Moreover, all three investigated specimens from Florida formed one highly supported cluster, however, only in the *COI*-2 trees.

**Fig 4:**
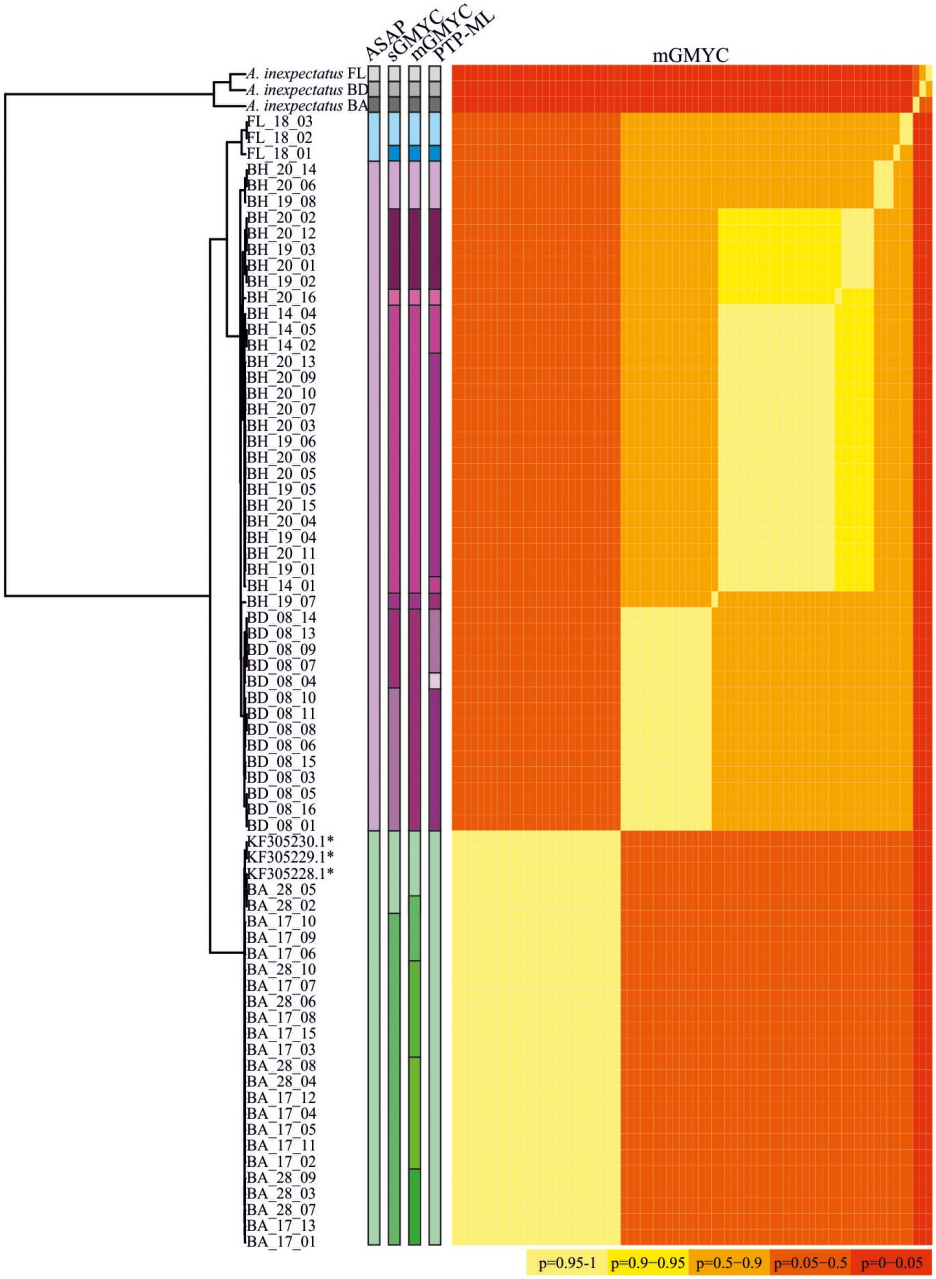
Ultrametric tree based on *COI*-2 sequence data. Results of five different SDAs are given on the right side.

Single SDA based on *COI*-2 data inferred 3–13 putative species, excluding outgroups (Fig 4): ASAP resulted in three different species, sGMYC in 11 putative taxa and mGMYC and PTP-ML in 13 species. Distance-based methods are congruent and identified the populations from Barbados and Florida each as a species and the populations from Bermuda and the Bahamas are given together as a single species. Tree-based methods identified numerous additional species, in some cases even within populations from a single location and thus clearly seem to have oversplit the species.

**Fig 5:**
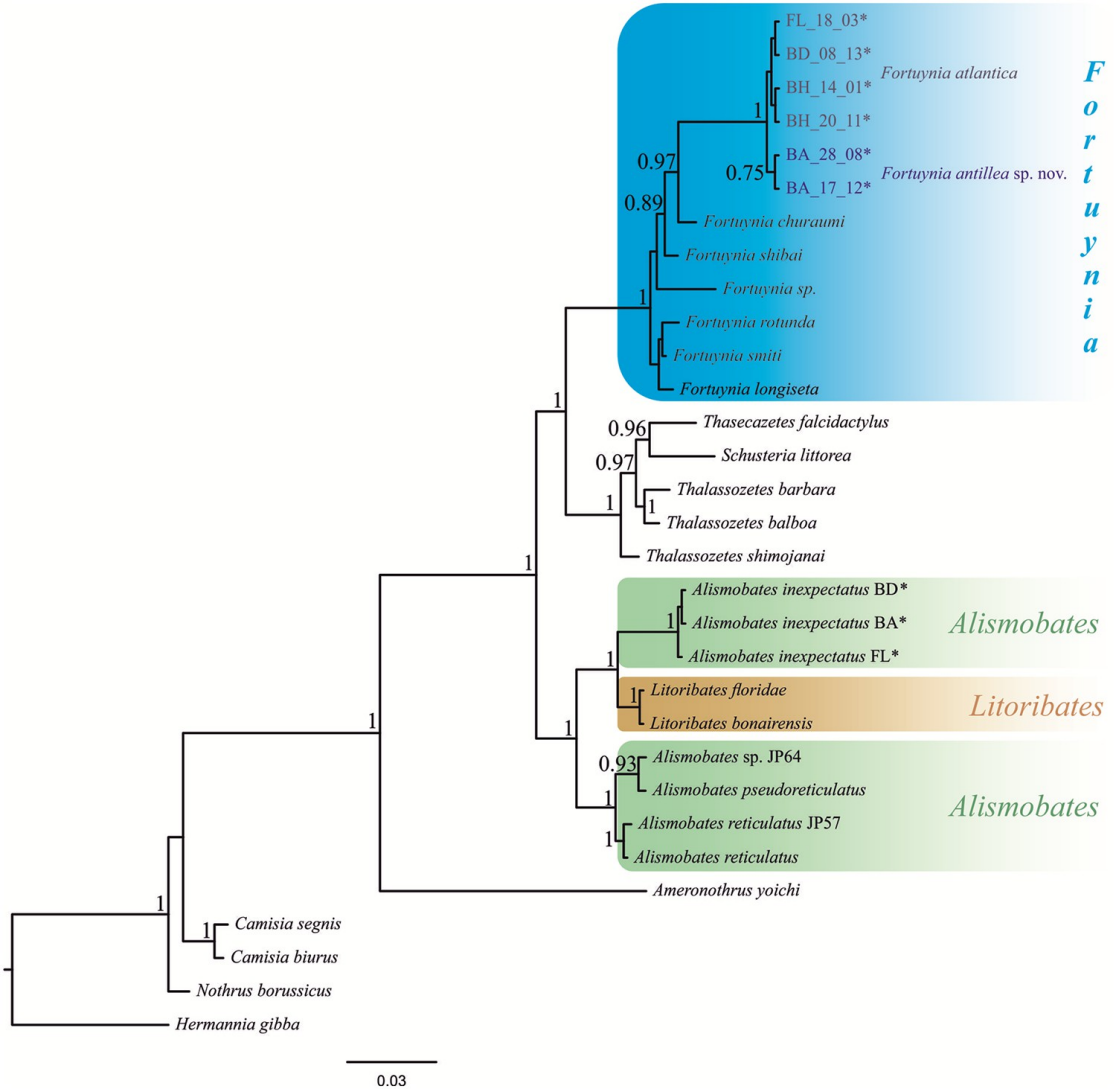
Bayesian inference tree based on *18S* gene sequences. Fortuyniid genera are shown in different colors; *indicates sequences generated in the present study, all other sequences were obtained from GenBank.

The p*18S* analyses (Fig 5) resulted in two different clades representing the Caribbean and Western Atlantic *Fortuynia*, one clade consisting only of Barbadian populations and the other clade comprising the populations from the Bahamas, Bermuda and Florida.

According to our results, the specimens from Barbados are given species rank and will from now on referred to as *Fortuynia antillea* sp. nov. (for details of this decision please see Discussion). Uncorrected pairwise distances in the *COI*-2 sequences between the two *Fortuynia* species were 9.9% and to the outgroup taxa distances ranged from 12.4% to 16.3% (Table 5).

**Table 5:** Mean uncorrected pairwise distances (a) between and (b) within the *Fortuynia* (*F*.) and *Alismobates* (*A*.) species investigated in this study. (a) Values for *COI*-2 are given in white boxes, respectively, *18S* in grey boxes. (b) n/c = not possible to estimate evolutionary distances because of insufficient sample size.

	Pairwise distances between species					Pairwise distances within species	
	*F*. *antillea* sp. nov.	*F*. *atlantica*	*A*. *inexpectatus* BD	*A*. *inexpectatus* FL	*A*. *inexpectatus* BA	*COI*-2	*18S*
*F*. *antillea* sp. nov.		0.001	0.029	0.029	0.029	0.001	0.000
*F*. *atlantica*	0.099		0.029	0.029	0.029	0.020	0.000
*A*. *inexpectatus* BD	0.162	0.146		0	0	n/c	n/c
*A*. *inexpectatus* FL	0.153	0.132	0.066		0	n/c	n/c
*A*. *inexpectatus* BA	0.168	0.146	0.097	0.099		n/c	n/c

The p*18S* phylogenetic tree depicts all investigated *Fortuynia* species as monophyletic group with the two species from the Caribbean and the Western Atlantic being a distinct clade within this group (Fig 5). The other fortuyniid genus *Alismobates* is given as paraphyletic taxon with one clade containing the Indo- and Western Pacific species and a second clade comprising the Caribbean specimens of *A*. *inexpectatus* with the Caribbean fortuyniid *Litoribates* species as sister group.

### Description of new species

Family Fortuyniidae Hammen, 1963

Genus *Fortuynia* Hammen, 1960

Type species: *Fortuynia marina* Hammen, 1960

*Fortuynia antillea* sp. nov. urn:lsid:zoobank.org:act:3605398C-18BE-4570-9298-6E7168DE1DCA

#### Type material

Holotype male (size 431μm x 256μm), Barbados: Bridgetown, Carlisle Bay (BA_17) brown short tomentose alga from quay wall, median eulittoral, 26 Feb. 2017. Paratypes (1 male 431μm x 250μm, 1 female 447μm x 269μm) same data as for holotype. All types deposited in the Senckenberg Museum für Naturkunde Görlitz (SMNG).

#### Etymology

The specific epithet ‘antillea’ refers to the island of Barbados (type locality) which is part of the Lesser Antilles.

#### Species diagnosis

**Fig 6:**
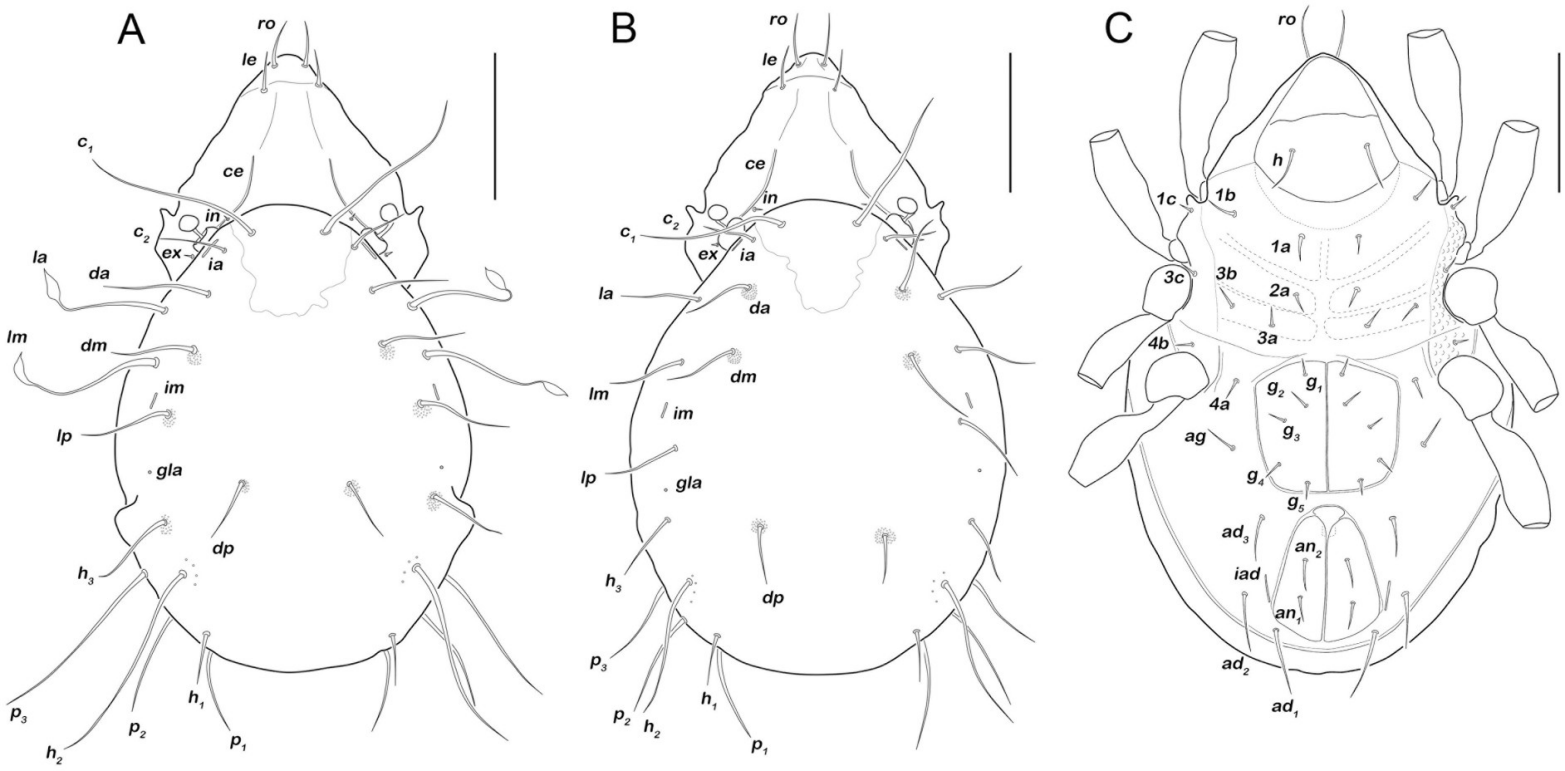
*Fortuynia antillea* sp. nov. morphology. A. Male dorsal view. B. Female dorsal view. C. Female ventral view (as there are no sexually dimorphic characters on the ventral aspect, apart from genital orifice size, this depiction is also valid for male). Scale bars = 100 μm.

Body length 406–439 μm. Sensillum smooth, short and clavate. Lamellar (*le*) and rostral setae (*ro*) of approximately the same length (Fig 6). One pair of prodorsal channels (*ce*) of medium length. Notogaster with 14 pairs of long setae, *c*_*3*_ absent. Strong sexual dimorphism present; males with long distally lanceolate setae *la* and *lm*, in females these setae setiform. Males with one obvious pair of lateral notogastral protuberances, females either lacking these protuberances or showing only faint protrusions. Epimeral setation 3-1-3-2, all setae short and spiniform, setae *3c* and *4b* in nearly lateral position close to the respective acetabulum. Five pairs of genital setae (*g*_*1*_-*g*_*5*_), one pair of aggenital setae (*ag*), three pairs of adanal setae (*ad*_*1-3*_) and two pairs of anal setae (*an*_*1*_-_*2*_).

#### Remarks

**Fig 7:**
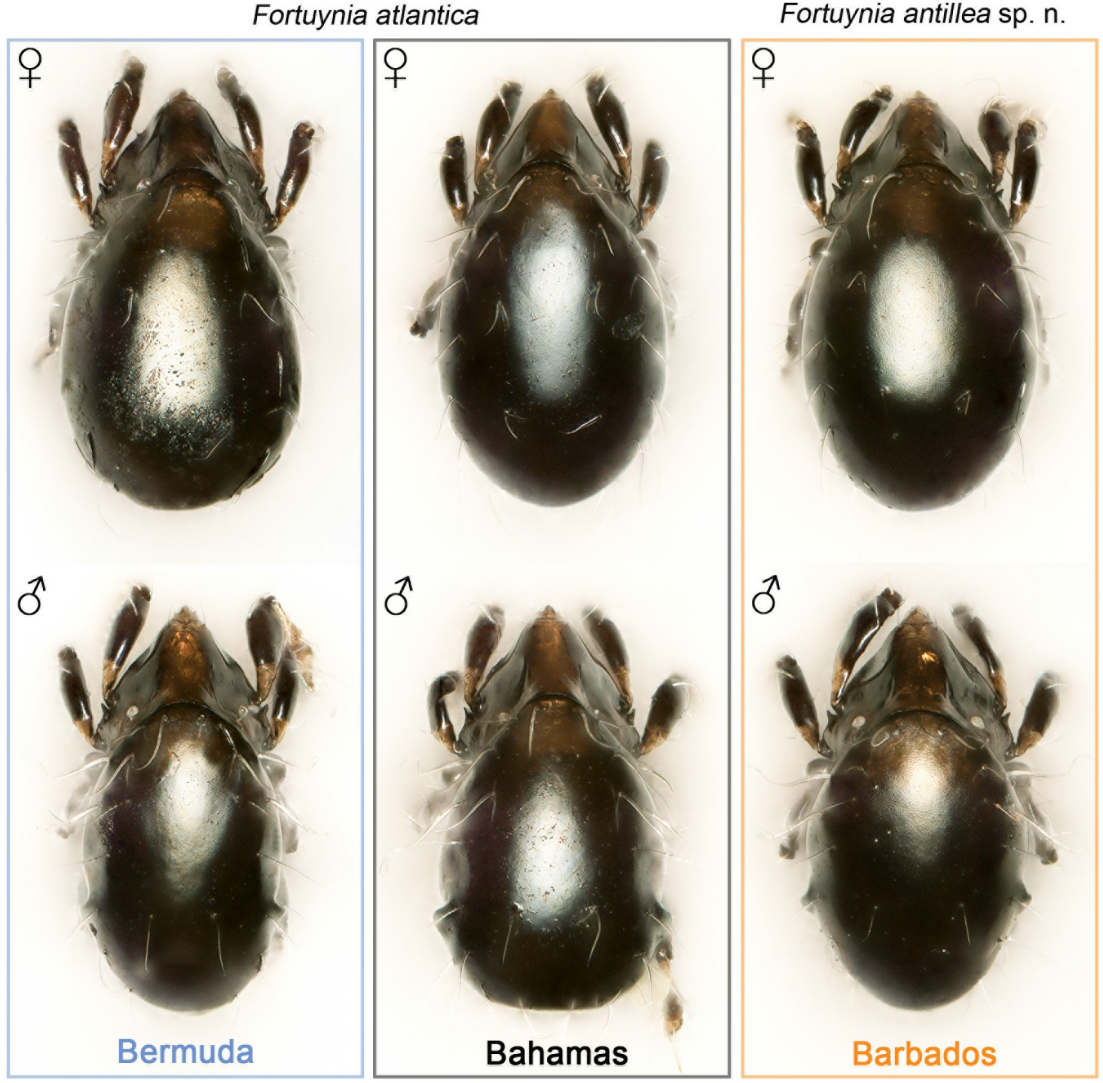
Photographic comparison of Caribbean *Fortuynia* species/populations from three different locations illustrating the lack of phenotypic differences. Stacked stereomicroscopic images in dorsal view; upper row female, lower row male specimens. (White spot on the notogaster is a light artifact / reflected light source).

The new species *F*. *antillea* sp. nov. shares the same phenotype with *F*. *atlantica* (Fig 7) and can thus not be distinguished based on microscopic investigation only. Therefore, we herein provide only the species diagnosis (which is also valid for *F*. *atlantica*) and no detailed description of each body part; the latter is provided by [7]. Consequently, species determination should always include the analysis of molecular genetic markers.

#### Diagnostic nucleotides

Nucleotide positions are relative and indicate the position within the herein used alignments. In *COI*-2, position 75 is occupied by base C, position 141 by base C, position 159 by base T and position 288 by base G. In the *18S* gene, position 190 is occupied by base G and position 201 by base G.

#### Distribution

Presently, *F*. *antillea* sp. nov. is only confirmed to be present on the Lesser Antillean island of Barbados. There are two other records of *Fortuynia* from the Lesser Antillean islands of Grenada and Guadeloupe but there were not enough specimens to confirm their taxonomic identity and thus it is yet impossible to tell if these occurrences can be attributed either to *F*. *atlantica* or to *F*. *antillea* sp. nov.

## Discussion

### Cryptic diversity

The Caribbean and Bermudian *Fortuynia* populations show a heterogeneous genetic structure which is in contrast to their homogeneous morphology. Distinct distinguishing morphological characters are absent and though morphometric data show a certain trend separating the island populations, no clear grouping can be observed that would allow to separate these populations on a morphological basis. Molecular genetic data, on the other hand, show strong divergences and certain deep splits that reveal a considerable diversity and phylogeographic structure in these intertidal *Fortuynia*. Cryptic diversity is not a rare phenomenon as it is known to occur in all biogeographic regions and in all major metazoan taxa [40]. Mites are no exception and there are numerous reports of cryptic species from diverse habitats and systematic groups [41]. In many cases, the reason for the morphological conformity is yet unknown, as for example in the cryptic diverse arboreal *Cymbaeremaeus*, where one supposedly easily recognizable species turned out to comprise eight genetically distinct species [42], or in the phoretic *Paraleius leontonychus*, which is suggested to contain six different cryptic species [36]. In intertidal mites, on the other hand, the morphological stasis is supposed to be a result of stabilizing selection caused by the extreme conditions prevailing in this environment [13]. The Caribbean intertidal *Carinozetes mangrovi* and *Carinozetes bermudensis*, for example, show wide distributions but consist of five distinct genetic lineages that cannot be distinguished based on morphology [13]. Another recent study [14] revealed the formerly widespread Caribbean intertidal mite *Thalassozetes barbara* to consist of seven morphologically identical but genetically distinct species whereas nearly all species represent island endemics. The present case of *Fortuynia* shows parallels and thus also highlights a correlation between cryptic diversity and the intertidal environment.

However, the absence of diagnostic morphological characters complicates the taxonomic evaluation of such genetically diverse groups and raises the question of how and when to delimitate species. Applying the biological species concept [43] is not feasible with these intertidal mites because cross-breeding experiments are very difficult to perform. *Fortuynia atlantica*, for example, is suggested to show mating behavior but hardly reproduced under laboratory conditions and mating behavior could never be observed so far [8]. Nevertheless, present species delimitation analyses based on genetic data resulted in at least two different Caribbean *Fortuynia* species. Some of the analyses resulted in even more taxa but those tree based-approaches are known to often oversplit species [44]. Genetic divergences based on the mitochondrial *COI* sequences between the two putative *Fortuynia* species were 9.9% and thus considerably exceed intraspecific divergence rates of other known oribatid mites, for example *Scutovertex minutus*, with 4% [45], *Steganacarus* spp. with <3.7% [46] or *Indopacifica pantai* with 6.4% [47]. Moreover, all individuals clustered congruently in the two single gene trees. In the cryptic Caribbean *Thalassozetes* species, on the other hand, the *18S* marker was not useful to separate the taxa with confidence [14] and even in the clear morphospecies *Fortuynia churaumi* and *F*. *shibai* from Japan this was not possible [48] due to incomplete lineage sorting. The two herein used markers thus clearly identify the *Fortuynia* from Barbados as a distinct lineage that is reproductively isolated from the other investigated populations. Given the genetic data, the *Fortuynia* populations from Barbados match the phylogenetic species concept and therefore we gave them species rank and named them *Fortuynia antillea* sp. nov. The specimens from Florida show also a distinct clade in the mitochondrial gene tree but we only studied very few individuals and they share the *18S* haplotype with the specimens from Bermuda. Therefore, we refrain from giving them species rank.

The three *Alismobates inexpectatus* specimens from different locations were also given as different taxa by all species delimitation analyses and uncorrected pairwise distances in the *COI* sequences ranged from 6.6 to 9.9% (see Table 5) and thus equal the divergence between the two *Fortuynia* species. Based on *COI* data only, they could also be assumed to represent different species but all share the same *18S* haplotype and the sample size is extremely low. A further study including more *Alismobates* specimens from all over the Caribbean is presently performed and will clarify if *A*. *inexpectatus* shows distinct genetic lineages or just higher intraspecific variation.

### Phylogeography

Based on molecular genetic data it is clear that *F*. *antillea* sp. nov. has been separated for a long time from the northern Caribbean *Fortuynia* populations and thus probably has evolved due to vicariance. At present, occurrences of *F*. *antillea* sp. nov. are only known from Barbados whereas it is unclear if this species represents a true island endemic. The island of Barbados is supposed to have emerged 1 my ago [49] but *F*. *antillea* sp. nov. is most likely older than this land mass considering an estimated molecular divergence rate of 2.15% per million years for the *COI* gene of oribatid mites [50]. Although this sounds paradox, this species could still be endemic to Barbados because such taxa or their ancestors, could have lived on former nearby islands that have been subsequently submerged or they have originally occurred on the mainland and later got extinct there [51]. However, there are records of *Fortuynia* from other Lesser Antillean islands, namely Grenada and Guadeloupe, and these occurrences may relate to *F*. *antillea* sp. nov. which would indicate a wider distribution of this species in the Lesser Antillean area. But unfortunately, genetic data of these specimens are lacking and thus they cannot be assigned to any of the known species. Due to this uncertain knowledge, it is not possible to answer if there is a clear biogeographic break between the northern Caribbean *F*. *atlantica* and the Lesser Antillean *F*. *antillea* sp. nov. or if there are any other evolutionary mechanisms responsible for the split-up of their ancestral species.

The genetic pattern of the northern Caribbean and Bermudian *F*. *atlantica* populations reflects geographic clusters but these clusters are rather diverse and do not represent distinct lineages, except for the specimens from Florida. Expansion and colonization events apparently have largely shaped their evolutionary history. The archipelago of Bermuda is a very small and remote oceanic landmass; accordingly, one would expect constant gene flow and thus homogeneous populations with little diversity, but we found eight different *COI* haplotypes existing at a single location. Due to the young geological age of Bermuda, i.e. the landmass rose above sea level ca. 900.000 years ago [52], it was already suggested that the *F*. *atlantica* populations originated from somewhere in the Caribbean area [9]. Genetic data show that Bermudian *F*. *atlantica* populations are closely related to northern Caribbean populations and thus confirm this geographic connection. Moreover, dispersal via the Gulf-Stream is supposed to be mainly responsible for the existence of several mites on Bermuda [6, 53] and genetic data now corroborates this hypothesis. The Gulf-Stream passes Florida and the northern Bahamas and flows northeastwards before it reaches Bermuda (see Fig 3). The Bermudian populations are most closely related to the Bahamian populations in terms of mitochondrial *COI* sequence data but they also share the same nuclear *18S* haplotype with the Florida specimens. Bird-mediated transport is suggested to be another possible mode of dispersal of flightless mites between islands [54] but most migratory birds overwintering in the Caribbean are following a clear north-south direction [55]. Moreover, the relatively short distance between Florida and the Bahamas can be easily travelled by even non-migratory birds but the *F*. *atlantica* populations from Florida and the Bahamas are genetically fairly distinct which contradicts bird-mediated dispersal, at least for this species. Darwin [56] already stated that ocean currents can also act as isolating factors and indeed, the Gulf-Stream flows rapidly through the gap between Florida and the Bahamas which may prevent animals to cross this gap by drifting on the water surface. This could explain why populations of the far distant Bermuda are closer related to the Bahamian populations while the latter are distinctly separated from the geographically close populations of Florida. Furthermore, the existence of numerous diverse *COI* haplotypes in the populations from Bermuda may be the result of different colonization events via the Gulf-Stream from the Bahamas region.

The *F*. *atlantica* populations from the small Bahamian New Providence Island show a strikingly similar pattern with even more haplotype diversity. The Bahamas Platform has been a stable carbonate block since the Cretaceous and reef development has kept it near sea level for much of that time [57]. But the low-lying islands have been created by accumulation and sedimentation by currents, waves and winds and most islands are dominated by Pleistocene rocks with a small amount of Holocene rocks [58]. Climate changes during the Pleistocene caused dramatic sea-level changes which means that landscape formation on the Bahamas Bank has been a very dynamic process with landmasses emerging and submerging during the last 2.5 mya years. This dynamic process seems to be reflected in the genetic data. *Fortuynia atlantica* populations on the Bahamas most likely have experienced tremendous changes due to colonization, expansion and extinction. Where the Bahamian populations originated from is yet unknown but most likely they are derived from ancestral populations from Hispanola and Cuba (Greater Antilles). *Fortuynia atlantica* is known to occur on the Greater Antilles [6] but genetic data is lacking so far. Therefore, the present results only provide a first insight into the phylogeography of Caribbean *Fortuynia* and inferred patterns should only be regarded as preliminary.

### Phylogeny of *Fortuynia*

The genus *Fortuynia* shows a transoceanic distribution with occurrences on subtropical and tropical coasts of nearly each continent [5]. The systematic relationships among members of different geographic regions have been unclear due to a remarkably homogenous morphology of all species [59] and due to missing molecular genetic data. Present molecular genetic results, based on *18S* sequences, point to a monophyletic origin of this genus with the Caribbean members being distinctly separated from the Indo-Pacific and Western Pacific species. However, the phylogeny is only based on a single genetic marker and less than half of the known *Fortuynia* species and therefore should only be regarded as preliminary data.

The genus *Alismobates*, as well as the family of Fortuyniidae, on the other hand, are given as paraphyletic taxa whereas the latter was already indicated by an earlier study [60]. In the present data, the Caribbean *Alismobates* is given as sister clade to *Litoribates* and these two clades are presented as sister group to the Indo- and Western Pacific *Alismobates*. The genus *Litoribates* is suggested to be closely related to *Alismobates* [61] and supposed to have evolved and diversified in the Caribbean region [6]. This is consistent with molecular genetic data and *Litoribates* may have evolved from Caribbean *Alismobates* stocks. However, the question remains why *Litoribates* diverged rapidly and considerably from *Alismobates* and why the latter not just diversified into more *Alismobates* species, as happened in other regions of the world. The answer could lie in the ecology of *Litoribates* because all known species are exclusive mangrove dwellers [6] whereas *Alismobates* members are either euryoecious or typical rock dwellers. Consequently, a Caribbean *Alismobates* ancestor may have adapted to mangrove environments which may have been accompanied by strong morphological divergences from the generic habitus and then the new genus further diversified within this environment.

Nonetheless, further morphological and molecular genetic data from all fortuyniid members are necessary to verify such a hypothesis.

## Supporting information

S1 Fig Bayesian inference trees based on A. *COI*-2 and on B. 18S gene sequences. (TIF)

S2 Fig Maximum likelihood tree based on 18S gene sequence. (TIF)

S3 Fig Photographs of genital orifice showing sexual dimorphism in size between male and female *F*. *atlantica*. (TIF)

S1 Table Specimens, GenBank accession numbers and sampling sites for the analyzed samples of the p*18S* dataset. (DOCX)

S1 Appendix Sample IDs, geographic origin, coordinates and GenBank accession numbers [numbers will be added upon acceptance] for *COI*, and *18S* sequences comprising all specimens included in genetic investigations. All specimens deposited in the collection of the Institute of Biology, University of Graz (IBUG).
(DOCX)
