# Supplementary figures and images for: A closer look reveals hidden diversity in the intertidal Caribbean Fortuyniidae (Acari, Oribatida)
Source: PLoS One. 2022 Jun 15;17(6):e0268964. doi: 10.1371/journal.pone.0268964 (PMC9200316; doi:10.1371/journal.pone.0268964)

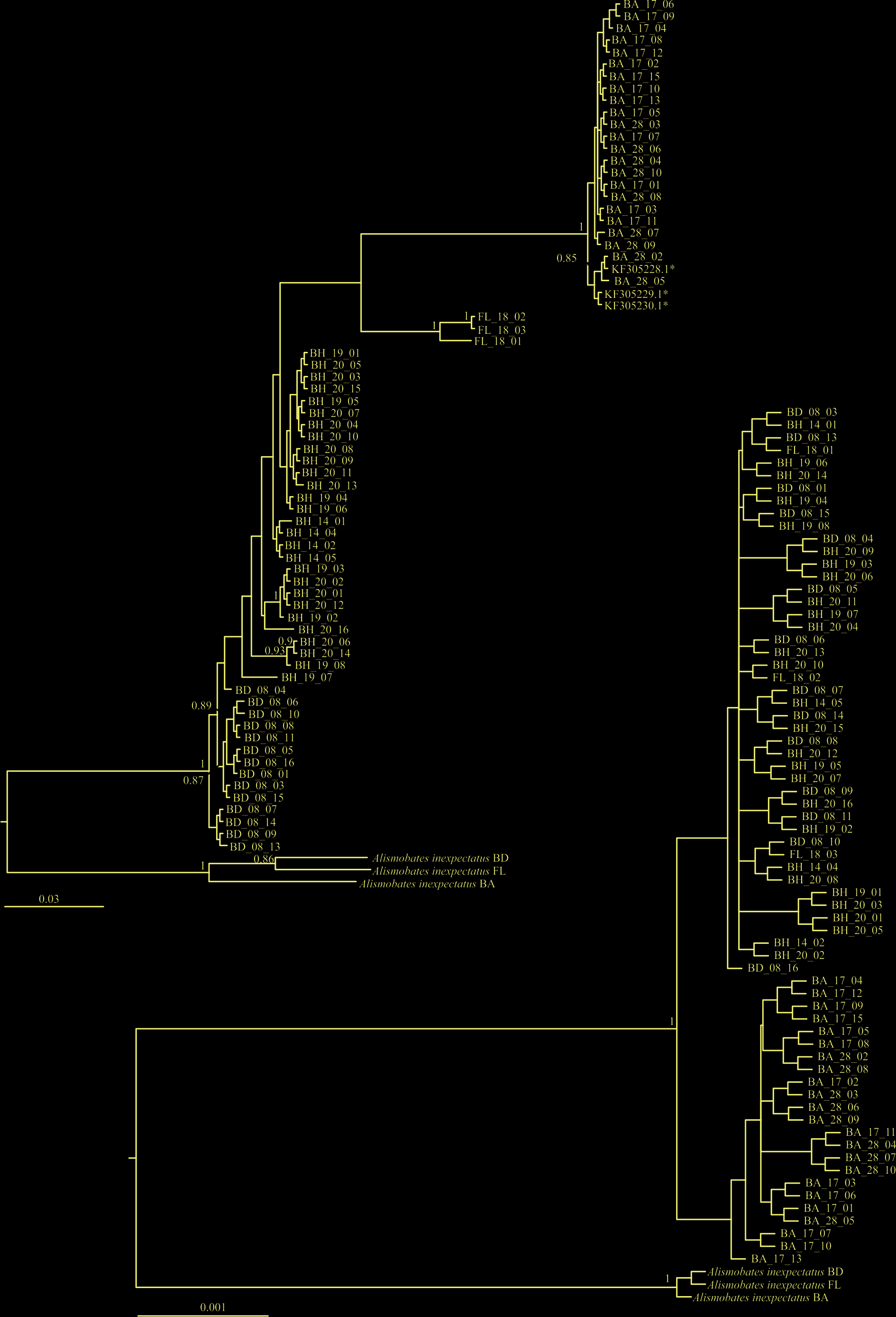

Supplement: S1 Fig — (TIF) [file pone.0268964.s001.tif]

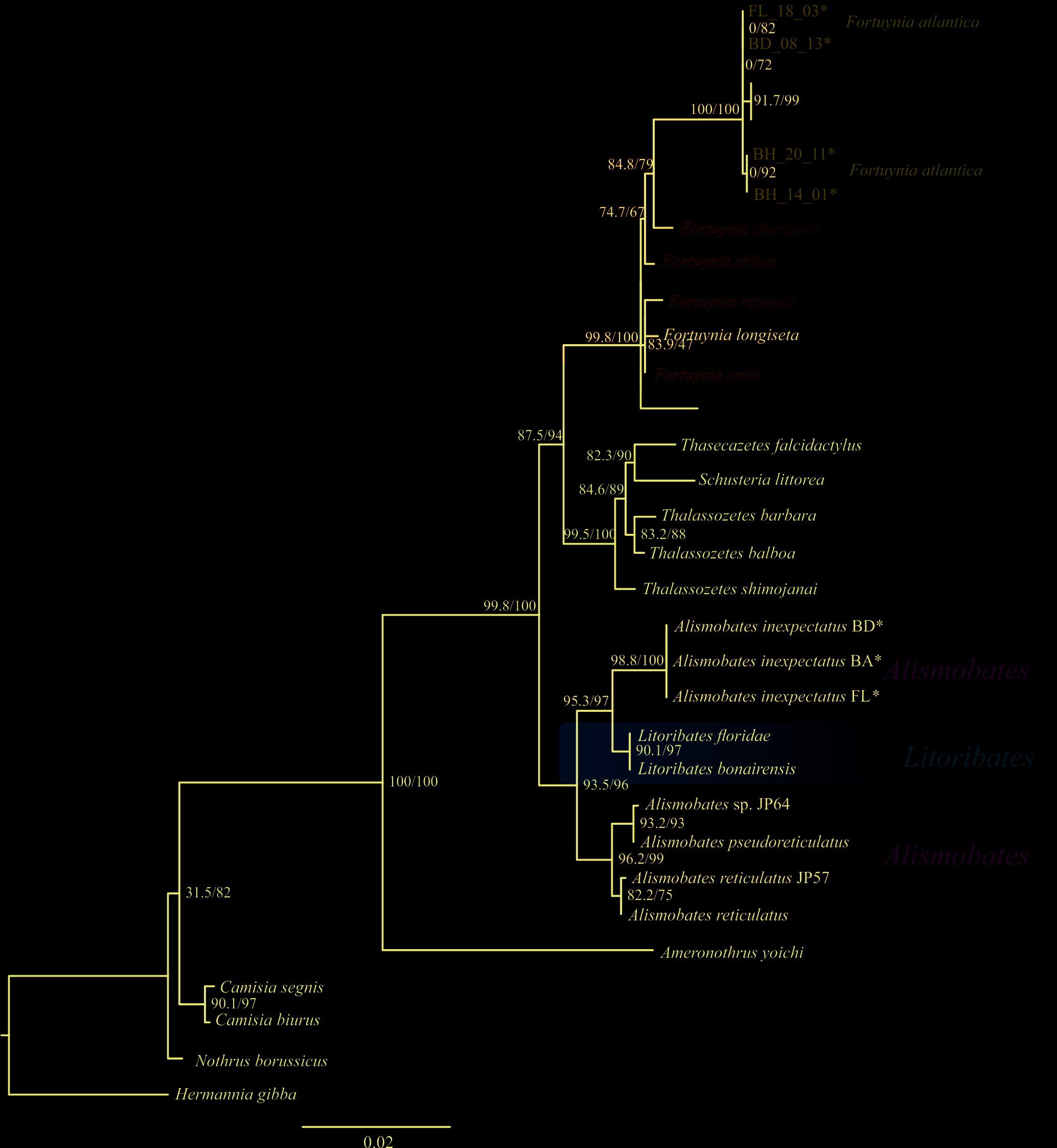

Supplement: S2 Fig — (TIF) [file pone.0268964.s002.tif]
