# Supplementary material for: A closer look reveals hidden diversity in the intertidal Caribbean Fortuyniidae (Acari, Oribatida)
Source: PLoS One. 2022 Jun 15;17(6):e0268964. doi: 10.1371/journal.pone.0268964 (PMC9200316; doi:10.1371/journal.pone.0268964)
Supplement: S1 Table — (DOCX) [file pone.0268964.s004.docx]

| Genus | Species / individual ID | GenBank Acc. Nr. | Sampling site |
| --- | --- | --- | --- |
|  |  |  |  |
| *Alismobates* | *pseudoreticulatus* | MH285696 | Malaysia |
|  | *reticulatus* JP57 | MN372418 | Japan |
|  | *reticulatus* | AB818526 | Japan Okinawa |
|  | sp. JP64 | MN372422 | Japan |
|  | *inexpectatus* BD* |  | Bermuda |
|  | *inexpectatus* FL* |  | Florida |
|  | *inexpectatus* BA* |  | Barbados |
| *Ameronothrus* | *yoichi* | MK88017 | Japan Hokkaido |
| *Fortuynia* | *antillea* sp. nov. / BA_17_12* |  | Barbados |
|  | / BA_28_08* |  | Barbados |
|  | *atlantica /* BH_14_01* |  | Bahamas |
|  | / BH_20_11* |  | Bahamas |
|  | / BD_08_13* |  | Bermuda |
|  | / FL_18_03* |  | Florida |
|  | *churaumi* | MN372424 | Japan |
|  | *longiseta* | MH285693 | Thailand |
|  | *rotunda* | AB818525 | Japan:Okinawa |
|  | *shibai* | MN372408 | Japan |
|  | *smiti* | MH285694 | Malaysia |
|  | sp. | MH285695 | Malaysia |
| *Litoribates* | *bonairensis* | MF997502 | Bonaire |
|  | *floridae* | MK035021 | Florida |
| *Schusteria* | *littorea* | HM070345 | Brazil |
| *Thalassozetes* | *balboa* | MK035019 | Panama |
|  | *barbara* | MK035020 | Barbados |
|  | *shimojanai* | AB818524 | Japan:Okinawa |
| *Thasecazetes* | *falcidactylus* | MF997501 | Bonaire |
|  |  |  |  |
| outgroup taxa |  |  |  |
| *Camisia* | *biurus* | EF081302 |  |
|  | *segnis* | JQ000043 |  |
| *Hermannia* | *gibba* | EF091426 |  |
| *Nothrus* | *borussicus* | KY922216 |  |

Table S1. Specimens, GenBank accession numbers and sampling sites for the analyzed samples of the p18S dataset.
