# Supplementary material for: A closer look reveals hidden diversity in the intertidal Caribbean Fortuyniidae (Acari, Oribatida)
Source: PLoS One. 2022 Jun 15;17(6):e0268964. doi: 10.1371/journal.pone.0268964 (PMC9200316; doi:10.1371/journal.pone.0268964)
Supplement: S1 Appendix — All specimens deposited in the collection of the Institute of Biology, University of Graz (IBUG). (DOCX) [file pone.0268964.s005.docx]

**Appendix.** Sample IDs, geographic origin, coordinates and GenBank accession numbers [numbers will be added upon acceptance] for *COI*, and *18S* sequences comprising all specimens included in genetic investigations. All specimens deposited in the collection of the Institute of Biology, University of Graz (IBUG)

|  |  |  |  |  | GenBank accession nr. | |
| --- | --- | --- | --- | --- | --- | --- |
| Country | Location | Sample ID | species | coordinates | COI | 18S |
| Barbados | Bridgetown | BA_17_01 | *F. antillea* sp. n. | 13.079329 -59.61255 | ON239327 |  |
|  |  | BA_17_02 |  |  | ON239328 |  |
|  |  | BA_17_03 |  |  | ON239329 |  |
|  |  | BA_17_04 |  |  | ON239330 |  |
|  |  | BA_17_05 |  |  | ON239331 |  |
|  |  | BA_17_06 |  |  | ON239332 |  |
|  |  | BA_17_07 |  |  | ON239333 |  |
|  |  | BA_17_08 |  |  | ON239334 |  |
|  |  | BA_17_09 |  |  | ON239335 |  |
|  |  | BA_17_10 |  |  | ON239336 |  |
|  |  | BA_17_11 |  |  | ON239337 |  |
|  |  | BA_17_12 |  |  | ON239338 | ON243683 |
|  |  | BA_17_13 |  |  | ON239339 |  |
|  |  | BA_17_15 |  |  | ON239340 |  |
|  | Bathsheba | BA_28_01 | *A. inexpectatus* | 13.062537 -59.541903 | ON239341 | ON243686 |
|  |  | BA_28_02 | *F. antillea* sp. n. |  | ON239342 |  |
|  |  | BA_28_03 |  |  | ON239343 |  |
|  |  | BA_28_04 |  |  | ON239344 |  |
|  |  | BA_28_05 |  |  | ON239345 |  |
|  |  | BA_28_06 |  |  | ON239346 |  |
|  |  | BA_28_07 |  |  | ON239347 |  |
|  |  | BA_28_08 |  |  | ON239348 | ON243682 |
|  |  | BA_28_09 |  |  | ON239349 |  |
|  |  | BA_28_10 |  |  | ON239350 |  |
| Bermuda | Whalebone Bay | BD_08_01 | *F. atlantica* |  | ON239351 |  |
|  |  | BD_08_02 | *A. inexpectatus* |  | ON239352 | ON243685 |
|  |  | BD_08_03 | *F. atlantica* |  | ON239353 |  |
|  |  | BD_08_04 |  |  | ON239354 |  |
|  |  | BD_08_05 |  |  | ON239355 |  |
|  |  | BD_08_06 |  |  | ON239356 |  |
|  |  | BD_08_07 |  |  | ON239357 |  |
|  |  | BD_08_08 |  |  | ON239358 |  |
|  |  | BD_08_09 |  |  | ON239359 |  |
|  |  | BD_08_10 |  |  | ON239360 |  |
|  |  | BD_08_11 |  |  | ON239361 |  |
|  |  | BD_08_13 |  |  | ON239362 | ON243679 |
|  |  | BD_08_14 |  |  | ON239363 |  |
|  |  | BD_08_15 |  |  | ON239364 |  |
|  |  | BD_08_16 |  |  | ON239365 |  |
| Bahamas | South Beach | BH_14_01 | *F. atlantica* | 25.001339 -77.350229 | ON239366 | ON243681 |
|  |  | BH_14_02 |  |  | ON239367 |  |
|  |  | BH_14_04 |  |  | ON239368 |  |
|  |  | BH_14_05 |  |  | ON239369 |  |
|  | Jaws Beach | BH_19_01 | *F. atlantica* | 25.018155 -77.546636 | ON239370 |  |
|  |  | BH_19_02 |  |  | ON239371 |  |
|  |  | BH_19_03 |  |  | ON239372 |  |
|  |  | BH_19_04 |  |  | ON239373 |  |
|  |  | BH_19_05 |  |  | ON239374 |  |
|  |  | BH_19_06 |  |  | ON239375 |  |
|  |  | BH_19_07 |  |  | ON239376 |  |
|  |  | BH_19_08 |  |  | ON239377 |  |
|  | Jaws Beach | BH_20_01 | *F. atlantica* | 25.018155 -77.546636 | ON239378 |  |
|  |  | BH_20_02 |  |  | ON239379 |  |
|  |  | BH_20_03 |  |  | ON239380 |  |
|  |  | BH_20_04 |  |  | ON239381 |  |
|  |  | BH_20_05 |  |  | ON239382 |  |
|  |  | BH_20_06 |  |  | ON239383 |  |
|  |  | BH_20_07 |  |  | ON239384 |  |
|  |  | BH_20_08 |  |  | ON239385 |  |
|  |  | BH_20_09 |  |  | ON239386 |  |
|  |  | BH_20_10 |  |  | ON239387 |  |
|  |  | BH_20_11 |  |  | ON239388 | ON243680 |
|  |  | BH_20_12 |  |  | ON239389 |  |
|  |  | BH_20_13 |  |  | ON239390 |  |
|  |  | BH_20_14 |  |  | ON239391 |  |
|  |  | BH_20_15 |  |  | ON239392 |  |
|  |  | BH_20_16 |  |  | ON239393 |  |
| Florida | Islamorada | FL_18_01 | *F. atlantica* | 24.9378169 -80.612182 | ON239394 |  |
|  |  | FL_18_02 |  |  | ON239395 |  |
|  |  | FL_18_03 |  |  | ON239396 | ON243678 |
|  |  | FL_18_04 | *A. inexpectatus* |  | ON239397 | ON243684 |
